# Association of Structural Global Brain Network Properties with Intelligence in Normal Aging

**DOI:** 10.1371/journal.pone.0086258

**Published:** 2014-01-22

**Authors:** Florian U. Fischer, Dominik Wolf, Armin Scheurich, Andreas Fellgiebel

**Affiliations:** Department of Psychiatry and Psychotherapy, University Medical Center Mainz, Mainz, Germany; Indiana University, United States of America

## Abstract

Higher general intelligence attenuates age-associated cognitive decline and the risk of dementia. Thus, intelligence has been associated with cognitive reserve or resilience in normal aging. Neurophysiologically, intelligence is considered as a complex capacity that is dependent on a global cognitive network rather than isolated brain areas. An association of structural as well as functional brain network characteristics with intelligence has already been reported in young adults. We investigated the relationship between global structural brain network properties, general intelligence and age in a group of 43 cognitively healthy elderly, age 60–85 years. Individuals were assessed cross-sectionally using Wechsler Adult Intelligence Scale-Revised (WAIS-R) and diffusion-tensor imaging. Structural brain networks were reconstructed individually using deterministic tractography, global network properties (global efficiency, mean shortest path length, and clustering coefficient) were determined by graph theory and correlated to intelligence scores within both age groups. Network properties were significantly correlated to age, whereas no significant correlation to WAIS-R was observed. However, in a subgroup of 15 individuals aged 75 and above, the network properties were significantly correlated to WAIS-R. Our findings suggest that general intelligence and global properties of structural brain networks may not be generally associated in cognitively healthy elderly. However, we provide first evidence of an association between global structural brain network properties and general intelligence in advanced elderly. Intelligence might be affected by age-associated network deterioration only if a certain threshold of structural degeneration is exceeded. Thus, age-associated brain structural changes seem to be partially compensated by the network and the range of this compensation might be a surrogate of cognitive reserve or brain resilience.

## Introduction

The main cognitive domains, including memory, attention, executive function, and information processing speed, are delimitable but not independent from each other. Individuals who perform well in one cognitive domain tend to do well in others. The common variance that is shared among cognitive domains is captured by the construct of general intelligence [1]–[3]. General intelligence has been shown to be a robust predictor of several life outcomes, including educational and occupational success, health-conscious behavior and mortality [1], [4], [5]. Moreover, general intelligence is known to attenuate the degree of age-associated cognitive decline and the risk of developing late-onset Alzheimer’s disease (AD) and has thus been proposed to be an important component of cognitive reserve [6]–[9].

In the last decade, neuroscientific research has strongly contributed to the understanding of the biological basis of human general intelligence. Quantitative genetic studies have shown basic genetic influences on intelligence [2], [10]. However, estimates of how much of the variance in general intelligence can be attributed to genetic variations range from 30 to 80% [2].

Besides genetic analyses, brain imaging studies contributed significantly to a better understanding of the biological basis of general intelligence. Structural and functional MRI studies have converged in identifying widespread parieto-frontal and temporal gray matter regions that were associated with intelligence [2], [11], [12]. Furthermore, there is evidence that intelligence relies on white matter connections between these gray matter regions forming a network that provides communication and integration of processes. [2], [13]–[15]. Thus structural brain networks may be the neurophysiological basis for general intelligence. Graph theory provides methods to describe and quantify organizational properties of these networks, which may have an impact on intelligence [16], [17].

Inspired by these findings, Li and colleagues reconstructed global structural brain networks in healthy young subjects using DTI tractography and assessed and quantified them with graph theoretical methods [14]. By demonstrating significant correlations between intelligence and network properties the authors provide first evidence for an association between the efficiency of brain structural networks and intelligence. Their findings are supported by another study by Zalesky and colleagues using a similar methodology as well as by a study investigating functional brain networks and intellectual performance by van den Heuvel and colleagues [18], [19].

Studies on normal aging demonstrated extensive WM degeneration accompanied by a reduction of network connectivity, exacerbated in old age [20]–[23]. Moreover, although general intelligence has been shown to be quite stable throughout the life-span [2], declines in intelligence scores with advancing age have repeatedly been reported [24]. Against the background of the observed association between the efficiency of brain structural networks and intelligence [14], age-related network alterations might contribute to age-related decline of general cognitive abilities, particularly in old age. However, investigations of the brain’s structural network and its relationship to general intelligence have not yet been applied to elderly. The current study was conducted to investigate age-related alterations of the global structural brain network, as measured by the combination of DTI tractography and MRI with graph analysis, and their effects on general intelligence in a group of younger and advanced, cognitively healthy elderly subjects.

## Materials and Methods

### Subjects

A sample of 43 cognitively healthy elderly aged 60 to 85 years had agreed to participate in the study that was conducted at the Department of Psychiatry and Psychotherapy, University Medical Center of Mainz, Germany. Subjects were recruited through advertisement in a local newspaper, as well as notices in medical practices and public institutions. The study has been approved by the local Ethics Committee of the Landesärztekammer Rheinland-Pfalz (state medical association of Rhineland-Palatinate) and all subjects gave written informed consent. Participants were living independently in the community and underwent a preceding psychiatric screening interview (DIA-SSQ) [25] in combination with International Diagnosis Checklists (IDCL) [26]. Subjects were excluded if they had suffered from any psychiatric, neurologic or cognitive disease prior to the study or if they were taking medication known to influence cognitive performance. All participants underwent comprehensive neuropsychological testing and Diffusion Tensor Imaging (DTI). Sample characteristics are shown in Table 1.

**Table 1:** Demographic characteristics and intelligence of the sample.

Age group	N (female/male)	Age	Years of education	WAIS-R IQ
Younger elderly (<75yrs)	28 (12/16)	65.2±4.1	13±3.7	139±15
Advanced elderly (≥75yrs)	15 (13/2)	79.1±3.6	10.9±3.0	136±17
Total sample	43 (25/18)	70.0±7.8	12.3±3.6	138±16
P-value	.006^a^*	<.001^b^*	.033^b^*	.990^b^

Mean ± standard deviation. Younger elderly: subjects aged 60 to 74. Advanced elderly: Subjects aged 75 to 85. P-value: a, chi-square test. b, Mann-Whitney test. *significant differences between groups, alpha = 0.05.

### Intelligence Scores

To assess general intelligence, we applied four subtests (Similarities, Arithmetic, Picture Completion, and Digit Symbol) of the revised Hamburg-Wechsler-Intelligence-Test (HAWIE-R) which is the German version of the Wechsler Adult Intelligence Scale-Revised (WAIS-R) [27]. This short form provides a reliable estimation of the Full Scale IQ [28].

### MRI Data Acquisition and Processing

Diffusion-weighted imaging was conducted on a Siemens 3T TrioTim MRI scanner (Siemens, Erlangen, Germany) using a single shot spin-echo echoplanar based sequence (30 directions; b = 1000 s/mm2; matrix 128×128; section thickness, 3mm; voxel size, 1.5×1.5×3 mm3; TR/TE, 7100 ms/102 ms). The data were corrected for subject motion and eddy currents using FSL 5.0 (FMRIB Analysis Group, Oxford, UK, http://www.fmrib.ox.ac.uk/fsl), gradients were adjusted accordingly [29]. Non-brain voxels were removed using FSL-BET [30]. A single diffusion tensor was fitted to each voxel using CAMINO v2 (Microstructural Imaging Group, University College London, UK, http://cmic.cs.ucl.ac.uk/camino/) and maps of the DTI index fractional anisotropy (FA) were calculated from its eigenvalues [31] for subsequent tractography. Additionally, two diffusion tensors were fitted [32], [33] to voxels likely containing fiber crossings as indicated by spherical harmonic fiber crossing detection [34].

### Reconstruction of the Individual Structural Brain Networks

#### Definition of the network’s nodes

The nodes of the structural brain networks were defined as the 111 cortical and subcortical anatomical brain areas in the Harvard-Oxford probabilistic brain atlas that is available with FSL 5.0 [35], [36]. In order to avoid overlapping between the brain areas, each area was thresholded at a probability of 0.35 and subsequently binarized [37].

To segment each subject’s brain in DTI space, DTI images were first coregistered to T1 images using FSL-FLIRT. The T1 images were then spatially normalized to MNI space using the DARTEL pipeline [38] in SPM8 (http://www.fil.ion.ucl.ac.uk/spm/software/spm8). Finally, the thresholded and binarized brain areas of the atlas were warped to DTI native space using the inverse transformations from DARTEL and the coregistration. The atlas brain areas thus transformed to each subject’s native DTI space were then used as the network nodes in the subsequent reconstruction of the structural network.

#### Reconstruction of the networks’ edges

**Figure 1:**
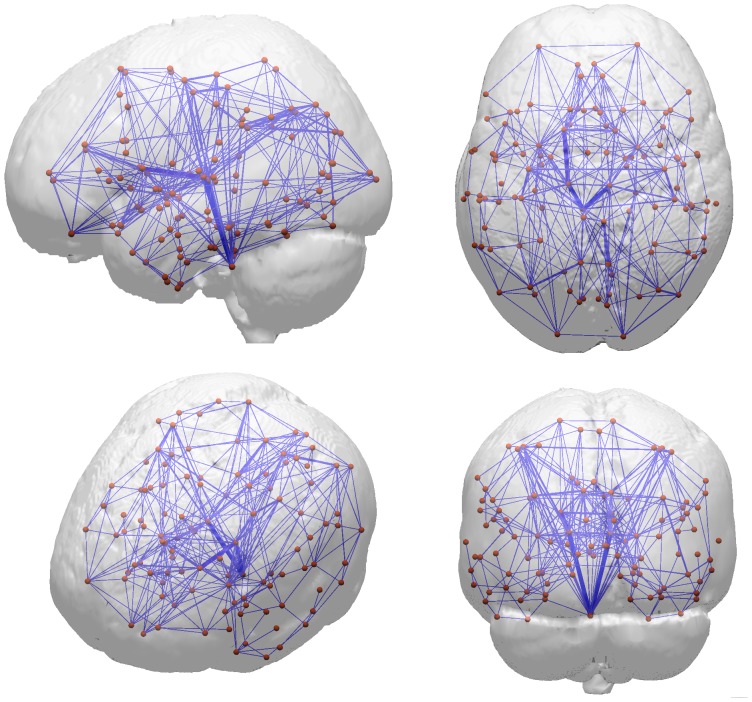
Structural network reconstructed in a male subject aged 73 years. Network nodes are shown as spheres that correspond to brain areas as defined by the Harvard-Oxford atlas. Connections are shown as blue lines, where wider lines indicate higher edge weights.

For this step whole brain tractography was performed in every subject using a deterministic streamline fiber tractography algorithm with a fixed step size as implemented in CAMINO. We have previously employed this algorithm successfully for the reconstruction of the bilateral cingulate bundles in Alzheimer’s Disease [39]. All voxels with FA ≥0.2 were used as seed points, FA and curvature thresholds were set to 0.2 and 60° respectively. Crossing fibers were taken into account by this tractography algorithm by considering two diffusion tensors in voxels that likely contain crossing fibers. Subsequently, for each pair of nodes, i.e. segmented brain regions from the atlas, those streamlines were extracted from the whole brain tractogram that intersect both of the two nodes. The number of the streamlines connecting two nodes were taken as the respective edge weight [14]. The resulting undirected, weighted brain graphs of each of the 43 subjects were represented as a 111 × 111 connectivity matrix *W^(i)^*, its entries *w^(i)^_pq_* containing the edge weight between nodes *p* and *q* in the brain graph of subject *i* respectively where *i* = 1 … *n, n* = 43 and *p,q* = 1 … 111. As the brain graphs are undirected, the 43 connectivity matrices *W^(i)^* are symmetric, i.e. *w^(i)^_pq_* = *w^(i)^_ qp_*_._ Self connections were set to zero with *w^(i)^_pq_* = 0 where *p* = *q*. Additionally, subsequent analyses were repeated for binarized networks that were calculated by setting all non-zero connection weights to one. Results are reported in Table S2. For a graphical representation of the resulting network, please see Figure 1.

Although tractography has been shown numerous times to reconstruct WM fiber pathways with reasonable accuracy [40], [41], the possibility of false positives remains in the reconstruction of connections between two brain areas. Therefore we chose to use a fixed threshold of three streamlines on the entries *w^(i)^_pq_* of each of the 43 subjects’ connectivity matrix as has been done before in brain graphs reconstructed from deterministic tractography [14], [42], [43]. To investigate the influence of the threshold choice, we repeated subsequent analyses with thresholds of 1, 2, 4 and 5 fibers per connection [14]. Results are shown as supplementary information in Table S1.

Additionally, we applied a rigorous statistical pruning algorithm that is based on the Holm-Bonferroni method to the brain graphs and has recently been proposed by Ivković et al. [44]. For each connection, the pruning algorithm attempts to refute the assumption that the distribution of connection weights across the sample has an expected value of zero. This is done by performing a z-test of the variance of the connection weights over the whole sample against a normal distribution centered at zero, whose variance is derived from all of the n matrices’ remaining non zero entries. If the assumption that both distributions are similar cannot be refuted, the connection is pruned in every subject’s graph by setting the corresponding *n* entries *w^(i)^_pq_* to zero. After testing all connections, the algorithm starts over. It will stop if none of the matrices’ entries have changed during the previous run. For each z-test, the p-value threshold was set to 0.001 [44]. Please note that the resulting brain graphs are still weighted and undirected and connections may be different across subjects, as some graphs may contain connections where other subjects’ graphs do not. Furthermore, the uncertainty in orientation of streamlines reconstructed by tractography is increased in voxels of lower FA and also accumulates in longer streamlines [45]–[47]. In order to take this uncertainty into account, we chose to calculate specific edge weights w^(i)*^_pq_ for the pruning procedure according to the formula 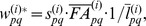 where 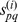 denotes the number, 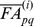 the mean FA and 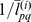 the inverse of the mean length in mm of the streamlines connecting node *p* and *q*.

### Quantification of the Brain Networks

The topological properties of the brain networks can be described using measures from graph theory. In this study, we chose to calculate clustering coefficient, mean shortest path length and global efficiency, as they have been show to be associated with intelligence in a previous study by Li et al. [14] as well as γ, λ and σ to report on the brain networks’ small world properties.

### Clustering Coefficient

The clustering coefficient measures the network's tendency to form dense local clusters and will be higher in networks with many such clusters. For weighted networks, there are several proposals for the calculation of the clustering coefficient [48]. In this study, we chose to calculate the clustering coefficient according to the extension proposed by Onnela et al. [49], as it takes the degree of the central node as well as all edge weights in the cluster into account. Thus the clustering coefficient of a node *r* was calculated as
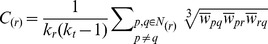
(1)where *k_r_* is the degree of node *r*, *N_(r)_* is the subset of all nodes directly connected to node *r* and 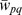 is the edge weight between nodes *p* and *q* normalized by division through the maximum edge weight in the subject's connectivity matrix [44]. For binary networks, the clustering coefficient was calculated as
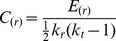
(2)Where E(r) is the set of all edges connected to nodes in N(r).

The clustering coefficient for each subject's brain graph was then calculated as
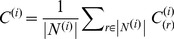
(3)where *N_(i)_* is the set of nodes in the network of subject *i*.

### Mean Shortest Path Length

This measure quantifies the shortest path length between each pair of nodes in the network. Higher weights and more connections will typically decrease the mean shortest path length. For the calculation of the shortest path between each pair of nodes, the distance associated with each edge was set to *d_pq_ = 1/w_pq_* [50]. The mean shortest path length for each subject was then calculated as.
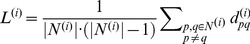
(4)


### Global Efficiency

Global efficiency is usually understood as a measure for the network's capacity to transfer information efficiently [51] and will be increased in a network with higher edge weights and shorter paths. Global efficiency is calculated from the shortest paths 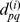 as
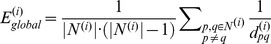
(5)


### Small Worldness

Small world attributes have been reported as a characteristic property of brain networks previously [16]. For the sake of comparability, we chose to evaluate them in our study as well. Networks are usually characterized as having small world properties if their clustering coefficients are higher than those of a random network, while their path lengths are comparable to that of a random network's. The small world indices σ, γ and λ were thus calculated as:
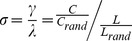
(6)


For each subject, 5000 random networks were constructed by randomly permuting the weights of the connectivity matrix while preserving its symmetry and keeping self-connections fixed as zero (Ivkovich und andere). Notably, the resulting random networks have the same number of edges, the same nodes and the same edge weights as the original networks, however edges will randomly connect different nodes. Subsequently, the clustering coefficient *C_rand_* and mean shortest path length *L_rand_* of the random network were then calculated as the average across the 5000 random permutations for each subject.

### Statistical Analysis

Partial correlations between the network measures and age were calculated in the total sample using years of education and gender as covariates. To investigate the relationship of intelligence scores and network measures, partial correlations were calculated in the total sample using age, years of education and gender as covariates.

In order to investigate whether the relationship of intelligence scores and network measures is altered in more advanced age, we additionally dichotomized the total sample into a group of younger elderly aged 60 to 74 years years and a group of advanced elderly aged 75 to 85 years (see Table 1 for demographical characteristics of the total sample and age groups). Age, years of education, intelligence scores and network measures were tested for normal distribution in both age groups using the Kolmogorov-Smirnov test. Additionally, homoscedasticity was ascertained using the Levene-test. We performed t-test for independent samples on normally distributed and non-parametric Mann-Whitney test on non-normally distributed variables to test for differences between the groups of younger and advanced elderly. Partial correlations between network measures and intelligence scores were calculated in the age groups as in the total sample using age, gender and years of education as covariates.

As there were only two men in the group of advanced elderly, gender was included as a covariate for all partial correlations. Additionally, we repeated the calculation of partial correlations between intelligence scores and network measures in the both age groups considering only the female subjects (Younger elderly, n = 12. Advanced elderly, n = 13).

The threshold for statistical significance for all tests was set to p = 0.05. To correct for multiple comparisons, the p-values were Holm-Bonferroni-corrected.

## Results

### Topological Properties of the Structural Brain Networks

We successfully reconstructed structural brain networks in each subject using a fixed connection threshold of at least 3 connecting fibers per edge and subsequent statistical pruning. The resulting networks showed overall small world characteristics for all subjects with a mean σ of 2.7±0.31, mean γ of 5.7±0.17 and mean λ of 2.1±0.23, although the path length was higher than in the random networks.

### Differences between Groups

Table 2 shows the absolute mean values and standard deviation of clustering coefficient, mean shortest path length and global efficiency. Global efficiency was decreased in the advanced elderly whereas mean shortest path length was increased. No significant group difference was found for the clustering coefficient.

**Table 2:** Comparison of network measures.

Age group	Clustering Coefficient	Mean Shortest Path Length	Global Efficiency
Younger elderly (<75yrs)	.0265±.0065	.0731±.0130	31.8±5.3
Advanced elderly (≥75yrs)	.0225±.0059	.0871±.0183	26.4±4.1
Total sample	.0251±.0065	.0780±.0163	29.9±5.5
P-value	.053	.006 **	.001 *

Mean values ± standard deviation. Younger elderly: subjects aged 60 to 74. Advanced elderly: Subjects aged 75 to 85. P-value: t-test for independent samples. Significant group differences after Holm-Bonferroni correction: * alpha = 0.05/3 = 0.017,** alpha = 0.05/2 = 0.025.

### Association of Network Measures with Age

While controlling for years of education and gender, a significant correlation with age was found in the total sample for the network measures clustering coefficient (p = 0.014), mean shortest paths (0.011) and global efficiency (0.033). The correlations remained significant after Holm-Bonferroni correction with alpha = 0.05/3 = 0.017, 0.05/2 = 0.025 and 0.05 respectively.

### Association of Network Measures and Intelligence Scores

**Figure 2:**
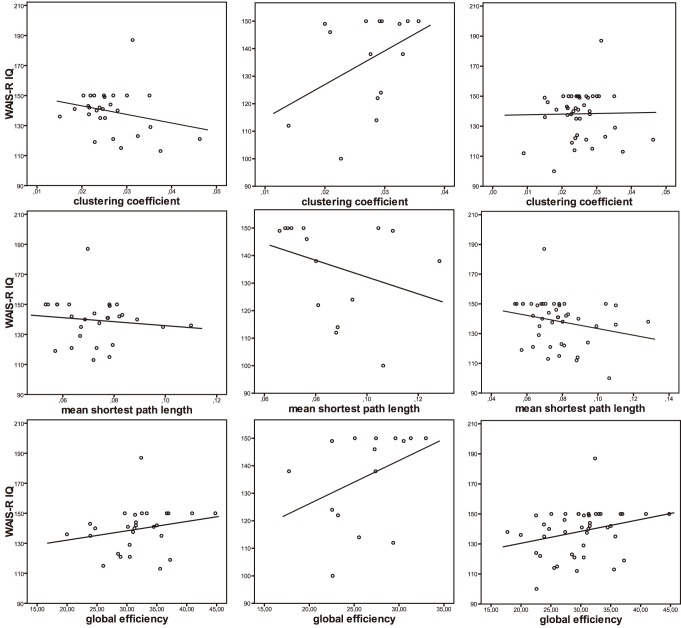
Results of partial correlation analyses investigating the association of network measures and intelligence scores. Left column: younger elderly (<75yrs). Middle column: advanced elderly (≥75yrs). Right column: total sample. Correlations for the advanced elderly were significant. Abbreviations: WAIS-R IQ, revised Wechsler Adult Intelligence Scale intelligence scores.

As shown in Table 3, no significant partial correlations of the network measures and intelligence scores were observed in the total sample or in the group of younger elderly. However, in the subgroup of advanced elderly significant correlations were observed, where a higher clustering coefficient and global efficiency as well as lower mean shortest path length were associated with higher intelligence scores. Notably, these results remain statistically significant after Holm-Bonferroni-correction. Please see Figure 2 for scatter plots and superimposed regression lines.

**Table 3:** Results of partial correlation analyses.

Age group	Network measure	WAIS-R IQ	
		PCC	p-value
	Clustering Coefficient	−.206	.323
Younger elderly (<75yrs)	Mean Shortest Path Length	.068	.746
	Global Efficiency	−.009	.966
	Clustering Coefficient	.752	.005*
Advanced elderly (≥75yrs)	Mean Shortest Path Length	−.721	.008**
	Global Efficiency	.589	.044***
	Clustering Coefficient	.066	.685
Total sample	Mean Shortest Path Length	−.229	.156
	Global Efficiency	.204	.208

Partial correlations of network measures and intelligence controlling for age, years of education and gender. Younger elderly: subjects aged 60 to 74. Advanced elderly: Subjects aged 75 to 85. WAIS-R IQ: The Wechsler Adult Intelligence Scale-revised (WAIS-R). PCC: partial correlation coefficient. Significant partial correlations after Holm-Bonferroni correction: *alpha = 0.05/3 = 0.017, **alpha = 0.05/2 = 0.025, ***alpha = 0.05.

Results of correlation analyses for networks calculated with connection weight thresholds 1 to 5 as well as binarized networks are shown in and S2 respectively.

### Additional Analyses

Considering only female subjects, the relationship of intelligence scores and network measures was unchanged compared to the main analysis after Holm-Bonferroni-correction. The p-values for clustering coefficient, mean shortest path length and global efficiency were in the total sample 0.457, 0.071 and 0.251 respectively. For the group of younger elderly they were 0.090, 0.619 and 0.563, for the group of advanced elderly they were 0.008, 0.010 and 0.049 respectively (respective p-value thresholds: alpha = 0.05/3 = 0.017, alpha = 0.05/2 = 0.025 and alpha = 0.05).

## Discussion

In this study we were able to successfully reconstruct weighted structural brain networks in 43 subjects aged 60 to 85 using DTI and deterministic fiber tractography. Small world properties were recorded for the networks and their topological properties were associated with age. Our most important finding was that structural brain networks' properties and intelligence (WAIS-R scores) were not correlated in the total sample, yet they were significantly correlated in a subsample of subjects aged 75 years and above. This finding suggests that an age-associated degeneration of network properties impacts on intelligence in advanced age.

### Topological Properties of the Networks

Small world properties could be demonstrated in various kinds of biological networks. Specifically, small world properties were found in human brain networks based on both structural and functional imaging data [16]. Likewise, the networks reconstructed in this study show some small-world properties such as σ = 2.7>1 and γ = 5.7 » 1. However, although λ is defined as ≈ 1 [17] we measured λ at 2.1 for the present networks. Increased λ has been reported in networks using the number of streamlines as edge weights before [14]. We think that the limitations of the tractography algorithm may be chiefly responsible for this result. As streamline deviations due to the uncertainty in streamline orientation accumulates [47], fewer streamlines may be reconstructed in longer WM pathways. However, long-range pathways connecting locally dense clusters are crucial for short paths in small world networks [52], thus lower edge weights in these long-range paths may lead to overall longer path length in the network. In confirmation of this view, after binarizing (setting all edge weights *w_pq_* >0 to 1) the networks reconstructed in this study, we measured λ at 1.3, which conforms to the definition of small-world attributes (data not shown).

### Changes of Network Measures with Aging

The network measures clustering coefficient and global efficiency were negatively correlated with age across groups. As global efficiency and mean shortest path length are inversely related, we accordingly found a positive correlation for the mean shortest path length with age. These findings show disconnections in the structural brain network that occur locally as well as globally and result in overall longer paths across brain regions. This was an expected finding, as the extensive WM degeneration that has been demonstrated in aging by several studies [53]–[56] implies significant alterations of the brain networks topological properties. Furthermore, Gong et al. were able to demonstrate age-related alterations of structural brain networks as well, showing both local and global alterations [23]. Yet they also found preserved global efficiency in aging. However, we do believe that this finding is not directly comparable to our data, as their sample included subjects of a different age range from 19 to 85 years.

### Changes of Intelligence Scores with Aging

Although general intelligence has been shown to be quite stable throughout the life-span [2], declines in intelligence scores with advancing age have repeatedly been observed [24]. In line with these results the group of younger elderly showed slightly higher intelligence scores than the group of advanced elderly in our study. However, this difference was not statistically significant. Of note, our study was based on a sample of homogeneous, highly intelligent subjects (mean WAIS-R IQ scores: younger elderly = 139; advanced elderly = 136). Higher levels of general intelligence have been shown to be related to lower levels of age-related cognitive decline [7], [8]. The high level of general cognitive abilities of both age groups in our study may have led to a rather slight age-related decline of intelligence scores and thus explain the lack of a statistically significant difference between both groups.

### Relationship between Intelligence and Network Measures

There is a growing consensus that intelligence is not based on a single narrowly defined region in the brain but rather on a group of frontal, parietal and temporal GM regions that have been identified using morphometry. These regions are connected by highly myelinated WM pathways and there is evidence that their integrity is related to cognitive ability. The view that intelligence is dependent upon a distributed network of brain areas has also been confirmed by functional MRI studies [2]. On the basis of this evidence, Li et al. have shown that topological properties of structural brain networks are associated with intelligence in a group of healthy young subjects [14].

The association between structural network properties and intelligence in the group of advanced elderly in this study are in agreement with these prior findings. Since intelligence emerges from a network structure of several interconnected brain areas, it seems reasonable that intelligence will be affected by disruptions of the network's connections that are more likely in a group of advanced elderly given the extensive WM degeneration in aging.

Interestingly, we did not find this association in the total sample or the group of younger elderly. A reasonable explanation for this finding is a threshold based relationship of the brain's structural network with intelligence – thus an effect on intelligence would only manifest once the network's topology has deteriorated beyond a certain point, at which inter-regional processing in the brain is sufficiently impaired. Since WM degenerates at an accelerating rate in aging [57], subjects in advanced age will more likely have structural networks deteriorated beyond the point at which effects on intelligence become manifest. However, the high level of intelligence scores in our sample implies very well connected structural networks. A sample consisting of subjects with average intelligence scores might exhibit structural networks closer to or below the presumed threshold such that network deterioration has a detectable effect on intelligence already at a younger age.

Furthermore, recent findings from fMRI indicate a reorganization of the brain's functional networks in normal aging [58]. It is reasonable to assume that this reorganization represents an attempt of functional compensation in the face of GM atrophy and dysfunction. Based on this assumption, the proposition of a threshold based relationship of the structural network with intelligence may be extended by proposing that functional compensation is dependent on a sufficient “infrastructure” provided by the structural network. If the structural network has deteriorated beyond a critical point, functional compensation may be impaired and effects on intelligence scores will be evident. Following this hypothesis, future studies may investigate the joint alterations of functional and structural brain networks in aging and their effect on cognition.

### Methodological Considerations/limitations

A number of methodological issues in this study need to be addressed. First, the deterministic tractography algorithm used in this study has been extended to take into account likely fiber crossings (see section MRI data acquisition and processing). However, it is possible that some connections between brain areas were missed. Second, even though we employed a rigorous statistical pruning method recently proposed, some false connections may remain in the reconstructed networks. Third, due to the cross sectional design of our study, potential cohort effects may have influenced results. Future studies should investigate the effects of longitudinal changes of structural brain networks on cognition. Fourth, tractography stopping criteria were fixed at values we had used before to successfully reconstruct major WM pathways in our data sample. As no gold standard has been established for tractography stopping criteria to date, other studies have used different parameters. Thus our results are of limited comparability with these studies. Fifth, the effect of outliers on the results of group comparisons of demographical characteristics may be underestimated due to the use of non-parametric tests. Sixth, the findings of this paper are confined to global structural network properties. Future studies should investigate network metrics quantifying specific local properties of structural networks and functionally defined subnetworks.

## Conclusions

In this study we investigated the association of global properties of structural brain networks and general intelligence in a group of cognitively healthy elderly aged 60 to 85 years. Interestingly, an association of network properties and general intelligence was not observed in the total sample. However, in a subgroup of subjects aged 75 years and above, we were able to demonstrate an association of age-related alterations of the network properties and general intelligence. More specifically, high local clustering and global efficiency as well as overall short paths between brain areas were correlated with higher intelligence scores. We therefore propose a threshold based relationship of structural brain network properties and intelligence. Network alterations that occur in aging may only have an effect on general intelligence once the characteristics of the network have deteriorated such that efficient communication and integrated processing between grey matter regions is impaired. Vice versa, the maintenance of intelligence seems to be independent from age-associated brain network degeneration to some extent. This individual “network buffering function” or “network compensation capability” might be a surrogate of cognitive reserve or brain resilience in normal aging which deserves to be further studied.

## Supporting Information

Table S1 **Results of partial correlation analyses for different connection weight thresholds.**
(DOC)

Table S2 **Results of partial correlation analyses of binarized, unweighted networks.**
(DOC)
