# Supplementary material for: Association of Structural Global Brain Network Properties with Intelligence in Normal Aging
Source: PLoS One. 2014 Jan 22;9(1):e86258. doi: 10.1371/journal.pone.0086258 (PMC3899224; doi:10.1371/journal.pone.0086258)
Supplement: Table S1 — Results of partial correlation analyses for different connection weight thresholds. (DOC) [file pone.0086258.s001.doc]

Table S1: Results of partial correlation analyses for different connection weight thresholds

| Threshold | Age group | Network measure | WAIS-R IQ | |
| --- | --- | --- | --- | --- |
|  |  |  | PCC | p-value |
|  |  | Clustering Coefficient | -.260 | .209 |
|  | Younger elderly (<75yrs) | Mean Shortest Path Length | .001 | .997 |
|  |  | Global Efficiency | -.001 | .997 |
|  |  | Clustering Coefficient | .776 | .003* |
| 1 | Advanced elderly (≥75yrs) | Mean Shortest Path Length | -.414 | .181 |
|  |  | Global Efficiency | .558 | .060 |
|  |  | Clustering Coefficient | .046 | .779 |
|  | Total sample | Mean Shortest Path Length | -.121 | .457 |
|  |  | Global Efficiency | .199 | .218 |
|  |  | Clustering Coefficient | -.246 | .235 |
|  | Younger elderly (<75yrs) | Mean Shortest Path Length | .007 | .973 |
|  |  | Global Efficiency | -.001 | .997 |
|  |  | Clustering Coefficient | .779 | .003* |
| 2 | Advanced elderly (≥75yrs) | Mean Shortest Path Length | -.594 | .042 |
|  |  | Global Efficiency | .573 | .051 |
|  |  | Clustering Coefficient | .058 | .721 |
|  | Total sample | Mean Shortest Path Length | -.254 | .114 |
|  |  | Global Efficiency | .207 | .199 |
|  |  | Clustering Coefficient | -.206 | .323 |
|  | Younger elderly (<75yrs) | Mean Shortest Path Length | .068 | .746 |
|  |  | Global Efficiency | -.009 | .966 |
|  |  | Clustering Coefficient | .752 | .005* |
| 3 | Advanced elderly (≥75yrs) | Mean Shortest Path Length | -.721 | .008** |
|  |  | Global Efficiency | .589 | .044*** |
|  |  | Clustering Coefficient | .066 | .685 |
|  | Total sample | Mean Shortest Path Length | -.229 | .156 |
|  |  | Global Efficiency | .204 | .208 |
|  |  | Clustering Coefficient | -.203 | .330 |
|  | Younger elderly (<75yrs) | Mean Shortest Path Length | -.016 | .938 |
|  |  | Global Efficiency | .009 | .967 |
|  |  | Clustering Coefficient | .749 | .005* |
| 4 | Advanced elderly (≥75yrs) | Mean Shortest Path Length | -.711 | .010** |
|  |  | Global Efficiency | .570 | .053 |
|  |  | Clustering Coefficient | .061 | .710 |
|  | Total sample | Mean Shortest Path Length | -.267 | .095 |
|  |  | Global Efficiency | .211 | .191 |
|  |  | Clustering Coefficient | -.198 | .344 |
|  | Younger elderly (<75yrs) | Mean Shortest Path Length | .027 | .896 |
|  |  | Global Efficiency | -.004 | .985 |
|  |  | Clustering Coefficient | .716 | .009* |
| 5 | Advanced elderly (≥75yrs) | Mean Shortest Path Length | -.651 | .022** |
|  |  | Global Efficiency | .545 | .067 |
|  |  | Clustering Coefficient | .051 | .755 |
|  | Total sample | Mean Shortest Path Length | -.208 | .199 |
|  |  | Global Efficiency | .194 | .231 |

Partial correlations of network measures and intelligence controlling for age, years of education and gender for different network weight thresholds. Younger elderly: subjects aged 60 to 74. Advanced elderly: Subjects aged 75 to 85. WAIS-R IQ: The Wechsler Adult Intelligence Scale-revised (WAIS-R). PCC: partial correlation coefficient. Significant partial correlations after Holm-Bonferroni correction: * alpha = 0.05 / 3 = 0.017, ** alpha = 0.05 / 2 = 0.025, *** alpha = 0.05.
