# Supplementary material for: Association of Structural Global Brain Network Properties with Intelligence in Normal Aging
Source: PLoS One. 2014 Jan 22;9(1):e86258. doi: 10.1371/journal.pone.0086258 (PMC3899224; doi:10.1371/journal.pone.0086258)
Supplement: Table S2 — Results of partial correlation analyses of binarized, unweighted networks. (DOC) [file pone.0086258.s002.doc]

Table S2: Results of partial correlation analyses of binarized, unweighted networks

| Age group | Network measure | WAIS-R IQ | |
| --- | --- | --- | --- |
|  |  | PCC | p-value |
|  | Clustering Coefficient | -.328 | .110 |
| Younger elderly (<75yrs) | Mean Shortest Path Length | .229 | .270 |
|  | Global Efficiency | -.262 | .206 |
|  | Clustering Coefficient | .494 | .103 |
| Advanced elderly (≥75yrs) | Mean Shortest Path Length | -.577 | .049 |
|  | Global Efficiency | .655 | .021 |
|  | Clustering Coefficient | -.052 | .749 |
| Total sample | Mean Shortest Path Length | -.071 | .663 |
|  | Global Efficiency | .081 | .617 |

Partial correlations of network measures of the binarized, unweighted networks and intelligence controlling for age, years of education and gender. Younger elderly: subjects aged 60 to 74. Advanced elderly: Subjects aged 75 to 85. WAIS-R IQ: The Wechsler Adult Intelligence Scale-revised (WAIS-R). PCC: partial correlation coefficient. Significant partial correlations after Holm-Bonferroni correction: * alpha = 0.05 / 3 = 0.017, ** alpha = 0.05 / 2 = 0.025, *** alpha = 0.05.
